# Surgery-related anxiety regarding arthroscopic meniscectomy under general anesthesia: a retrospective observational study

**DOI:** 10.1186/s12891-023-07112-3

**Published:** 2023-12-19

**Authors:** Chae-Chil Lee, Jae-Ryong Cha, Jang-Ho Park, Min-Seok Kim, Ki-Bong Park

**Affiliations:** 1 Department of Orthopedic Surgery, Ulsan University Hospital, University of Ulsan College of Medicine, 25 Daehakbyeongwon-ro, Dong-gu, Ulsan, 44033 Republic of Korea; 2 Department of Psychiatry, Ulsan University Hospital, University of Ulsan College of Medicine, Ulsan, Republic of Korea

**Keywords:** Knee, Arthroscopy, Meniscectomy, Anesthesia, Anxiety

## Abstract

**Background:**

The prevalence of anxiety among patients undergoing arthroscopic surgery and its association with postoperative function has been well documented; however, the level of anxiety and anxiety-related characteristics remain unclear. As such, the present study investigated the characteristics of state anxiety in patients undergoing arthroscopic meniscectomy.

**Methods:**

Data from 75 patients, who underwent arthroscopic partial meniscectomy under general anesthesia and completed an anxiety status questionnaire between April 2021 and March 2022, were retrospectively collected and reviewed. The State-Trait Anxiety Inventory (STAI)-X was used to measure state anxiety; a total score ≥ 52 was defined as clinically meaningful state anxiety. STAI score, main cause of preoperative anxiety, most anxious period, and most helpful factors for reducing perioperative anxiety were investigated. Patients were divided into 2 groups according to the main cause of preoperative anxiety; surgery or anesthesia (group I [n = 47]); and postoperative pain or rehabilitation (group II [n = 28]) Characteristics of state-anxiety between the two groups were compared using independent *t*-tests.

**Results:**

The mean STAI score of the total population was 39.1 points (range, 20–60 points). The mean STAI score was significantly higher in group I than in group II (41.9 vs. 34.4 points, respectively; *P* < 0.001). The proportion of patients with clinically meaningful state anxiety was significantly higher in group I than in group II (23.4% vs. 3.6%, respectively, *P* = 0.02). Most patients (66.0% in group I and 50.0% in group II) responded that trust in medical staff was the most helpful factor in overcoming preoperative anxiety. In group I, 63.8% reported that the surgeon’s explanation was the most helpful factor in reducing postoperative anxiety, whereas in group II, 71.4% reported that the natural course after surgery was the most helpful factor.

**Conclusions:**

Surgeons should be aware that anxiety related to arthroscopic meniscectomy differs according to patient characteristics, and a preoperative explanation of the postoperative process with the surgeon is important for patients who experience preoperative anxiety regarding anesthesia or the surgery itself.

## Background

Arthroscopic meniscectomy is one of the most common orthopedic procedures [1, 2]. In general, physicians explain to patients that arthroscopic meniscectomy is simple, the operative duration and hospitalization period are short and few major postoperative complications are encountered [3, 4]. However, patients complain of anxiety regarding the surgery or general anesthesia, including fear of death, inability to wake up after surgery, feeling pain during surgery, and awakening during surgery [5].

Several studies investigating the effects of anxiety or positive psychological responses on functional outcomes after arthroscopic surgeries on the shoulder joint have been performed [6–10]. Similarly, in patients undergoing arthroscopic meniscectomy, studies are needed to evaluate the effect of psychological status on functional outcomes by ascertaining the level and main causes of preoperative anxiety [11–13]. However, the prevalence of clinically meaningful state anxiety, patient characteristics that can affect the level of anxiety, the main cause of anxiety, the most anxious period, and resolution factors are currently unclear. As such, the present study investigated the levels and main causes of preoperative anxiety in patients who underwent arthroscopic meniscectomy. We hypothesized that the methods used to reduce anxiety would differ depending on the main cause of surgery-related anxiety.

## Methods

### Study design

This retrospective observational study recruited subjects who underwent arthroscopic partial meniscectomy under general anesthesia between April 2021 and March 2022.

### Subjects

Surgical treatment was performed in patients whose symptoms and signs were not controlled despite receiving conservative treatment consisting of medication and physical therapy for at least three months. Patients who elected to undergo surgical treatment underwent rehabilitation education, including active range of motion and quadriceps muscle strengthening exercises, which should be performed postoperatively. A single surgeon specializing in operative procedures for the knee joint performed all surgeries. At the author’s institution, general anesthesia is routinely administered to patients undergoing arthroscopic meniscectomy. Patients are discharged on the day after surgery and undergo rehabilitation treatment while attending the hospital 2–3 times a week. Muscle strengthening exercises are recommended until quadriceps strength on the affected side recovers to ≥ 80% of that on the unaffected side.

### Inclusion and exclusion criteria

Patients who were ≥ 19 years of age and completed the questionnaire were included in the study. Individuals with a history of orthopedic surgery or psychiatric disorders, those who underwent arthroscopic meniscal repair due to acute traumatic meniscal tears, those who underwent microfracture for concomitant lesions of the articular cartilage, and those who precluded recruitment and investigation due to emergent arthroscopic synovectomy for septic arthritis were excluded.

### State-anxiety assessment and questionnaire

The primary outcome was the State-Trait Anxiety Inventory (STAI) score used to measure anxiety levels. The STAI-X, which consists of 20 questions in total, was used [14]. The scores for each question range from 1 to 4; thus, the total score for each scale ranges from 20 (lowest) to 80 (highest), with higher scores indicating higher levels of anxiety. A total score ≥ 52 indicates clinically meaningful anxiety. Perioperative anxiety related to arthroscopic meniscectomy was evaluated using a questionnaire introduced in previous studies [15, 16]. The questionnaire was divided into four areas: main cause of anxiety; most anxious moment during the entire process; most helpful factors for overcoming preoperative anxiety; and reducing postoperative anxiety.

### Subgroup analysis

Responses to the questionnaire addressing perioperative anxiety were analyzed, and subjects were divided into 2 groups according to the main cause of anxiety. Subjects who responded that the surgery itself or the anesthesia caused their preoperative anxiety were assigned to group I, while those who responded that the pain or rehabilitation they would experience after surgery caused their preoperative anxiety were assigned to group II. The STAI score, proportion of clinically meaningful state anxiety, most anxious period, most helpful factors in overcoming preoperative anxiety, and reducing postoperative anxiety were also analyzed.

### Statistical analysis

All measures are expressed as mean (range). Normal distribution of data was checked by descriptive measures such as coefficients of skewness and kurtosis. Independent *t*-tests were performed using SPSS (IBM Corporation, Armonk, NY, USA) for Windows version 11.5. Differences with *P* < 0.05 were considered to be statistical significant.

### Ethics approval

The present study was approved by the institutional review board of the author’s institution and informed written consent was obtained from all subjects.

## Results

Seventy-five subjects were included in the final analysis, of whom 46.7% (n = 35) were female, with a mean age of 53.4 years (range, 46–63 years). A summary of the demographic characteristics of subjects is shown in Table 1 and all variables were normally distributed. None of these subjects were professional athletes and all developed meniscal tears due to non-professional injuries. The 75 subjects were divided in 2 groups; group I, n = 47 (62.7%); and group II, n = 28 (37.3%).

The proportion of female and mean age were significantly higher in group I than in group II (Table 1). The odds ratios (ORs), which indicated the probabilities of experiencing anxiety regarding the surgery itself or anesthesia, were 8.1 for females compared with males, and 6.8 for subjects ≥ 55 years of age compared with those ≤ 55 years of age. The mean age of the 35 female and 40 male subjects was 53.2 and 53.6 years, respectively, with no significant difference (*P* = 0.71). There were no statistically significant differences in height, weight, or body mass index between the groups.

**Table 1:** Demographic characteristics of subjects who underwent arthroscopic partial meniscectomy

Variable	Group I (n = 47)	Group II (n = 28)	*P*-value
Female sex	30 (63.8)	5 (17.9)	< 0.001
Age, years	54.3 (46–63)	51.9 (46–57)	0.01
≥55	27 (57.4)	8 (28.6)	
<55	20 (42.6)	20 (71.4)	
Height, cm	159.9 (150.0–173.0)	160.1 (149.6–173.6)	0.26
Weight, kg	63.1 (52.2–77.8)	63.9 (52.0–80.0)	0.11
Body mass index, kg/m^2^	24.6 (22.8–28.3)	24.8 (22.1–29.2)	0.48

Data presented as n (%) or mean (range) unless otherwise indicated

The mean STAI score of the total population was 39.1 points (range, 20–60). The mean STAI score was significantly higher in group I (41.9 points) than in group II (34.4 points, *P* < 0.001) (Table 2). The proportion of patients with clinically meaningful state anxiety was significantly higher in group I (23.4%) than in group II (3.6%) (*P* = 0.02).

**Table 2:** Mean State-Trait Anxiety Inventory score and proportion of subjects with clinically meaningful state-anxiety

Variable	Group I	Group II	*P*-value
State-Trait Anxiety Inventory score, mean (range)	41.9 (20–60)	34.4 (23–56)	< 0.001
Subjects with clinically meaningful state-anxiety^*^, n (%)	11 (23.4)	1 (3.6)	0.02

^*^ State-Trait Anxiety Inventory score ≥ 52

Most patients in both groups (group I, 93.6%;group II, 71.4%) responded that they were most anxious during the period from admission to surgery. During the wait period after deciding on surgery and admission, 25.5% and 10.7% of patients in groups I and II, respectively, reported that they were most anxious. Conversely, 6.4% and 28.6% of patients in groups I and II, respectively, were most anxious during the hospitalization period from surgery to discharge (Table 3).

**Table 3:** Moment of the highest level of anxiety in the perioperative period

Group	OPD-ADM	ADM-AS	AS-DC
I	12 (25.5)	32 (68.1)	3 (6.4)
II	3 (10.7)	17 (60.7)	8 (28.6)

OPD, outpatient department; ADM, admission; AS, arthroscopic surgery; DC, discharge

**Fig. 1:**
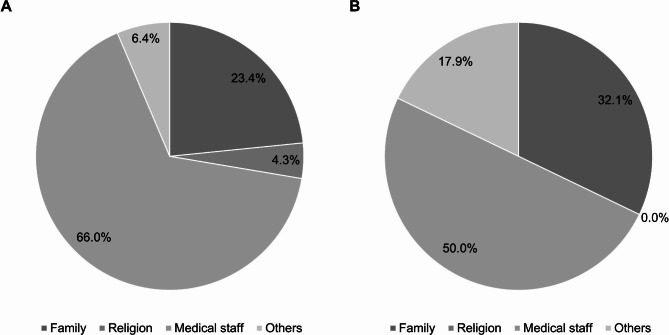
Most helpful factors in overcoming anxiety before arthroscopic partial meniscectomy **(a)** Group I; **(b)** Group II

Most patients in both groups (group I, 66.0%; group II, 50.0%) responded that trust in medical staff was the most helpful factor in overcoming preoperative anxiety (Fig. 1).

**Fig. 2:**
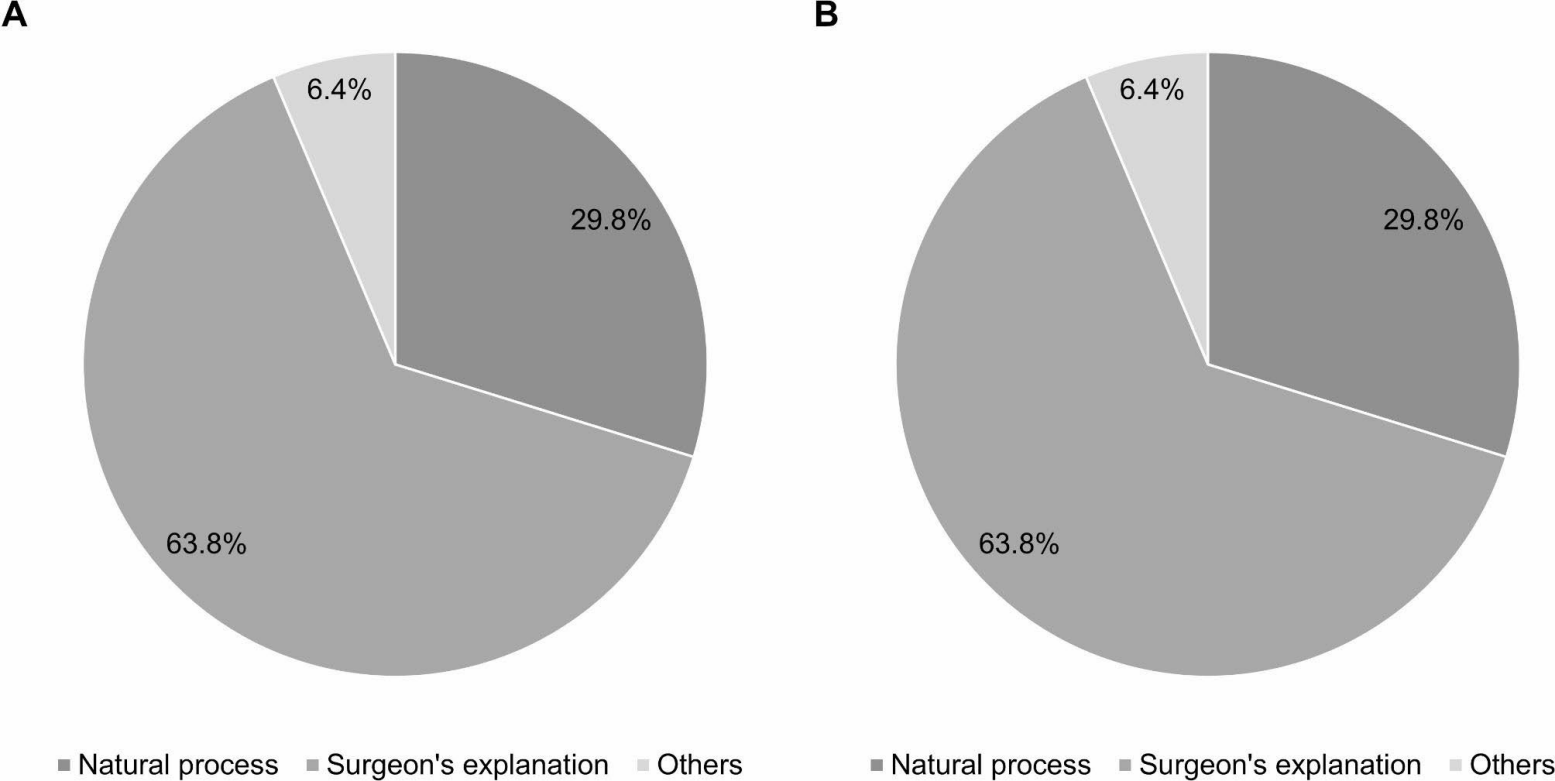
Most helpful factors in reducing anxiety after arthroscopic partial meniscectomy. **(a)** Group I; **(b)** Group II. Natural process included the conclusion of surgery, and symptoms improved

Most patients (63.8%) in group I responded that the surgeon’s explanation of the surgery was the most helpful factor in reducing postoperative anxiety, whereas 71.4% of patients in group II responded that the natural course, such as completion of surgery or improvement of symptoms, was the most helpful factor (Fig. 2).

## Discussion

Results of the present study demonstrated that female sex and advanced age were risk factors for anxiety regarding the surgery itself or anesthesia, and the most helpful factor in reducing postoperative anxiety differed according to the content of the patient’s anxiety regarding arthroscopic partial meniscectomy.

### STAI score

Data regarding anxiety levels in patients undergoing arthroscopic meniscectomy are lacking. Cornoiu et al. [17] reported that the mean STAI score of patients who underwent arthroscopic knee surgery was 33.0 points. Tharion and Kale [18] analyzed 98 patients who underwent arthroscopic knee surgery and reported that the median (25th − 75th percentile) preoperative STAI scores ranged from 26.7 to 46.7 points. Yang et al. [13] used the Amsterdam Preoperative Anxiety and Information Scale score to measure the level of anxiety in patients undergoing arthroscopic knee surgery and reported that the median combined anxiety component scores were 4.0–8.5 in the virtual reality group and 5.3–9.8 in the control group. In this study, the mean STAI score was 39.1 (range, 20–60).

### Risk factors for preoperative anxiety

Previous studies have reported that female sex, advanced age, and high educational level are risk factors for preoperative anxiety [5, 19, 20]. The present study revealed that female sex and advanced age were risk factors for anxiety regarding the surgery itself or the anesthesia. Therefore, surgeons should recognize that these patients are anxious about surgery, and should empathize and provide sufficient information regarding anesthesia.

### Most anxious period

Several studies have investigated the period of greatest anxiety in patients who have undergone surgery other than arthroscopic knee surgery [15, 16]. However, there are no studies investigating the period of greatest anxiety in patients who undergo arthroscopic knee surgery, except for 2 that reported changes in STAI scores before and after the procedure. Tharion and Kale [18] compared anxiety levels before and after arthroscopic knee surgery and reported that STAI scores after surgery were 0–16.7 points lower. Bayar et al. [11] reported a significant decrease in the STAI-S score postoperatively in the experimental group and in those watching their arthroscopy. Although the level of anxiety before and after surgery could not be compared in this study, as in previous studies, the period when anxiety was most severe was investigated, which revealed that 70–90% of patients responded within the period from admission to surgery. Therefore, it can be assumed that the subjects in this study may have had decreased postoperative anxiety scores compared to their preoperative anxiety scores, as in previous studies.

### Demand for information related to surgery or anesthesia

Several studies have reported a correlation between patient level of anxiety related to surgery or anesthesia and the demand for such information. Jarmoszewicz et al. [20] reported that patients with high preoperative anxiety also had a high desire for information, and confirmed that patient need to be informed was present at the stage of decision-making regarding surgery. The literature reports that preoperative information or explanations of the surgery reduce anxiety and increases satisfaction [21, 22]. Sahin and Basak [23] emphasized that good communication skills may reduce patient STAI scores, and when patients are informed about the procedure and why it is being performed, their anxiety levels may decrease. Bayar et al. [11] conducted a prospective randomized study to determine the effect of watching simultaneous arthroscopic views on postoperative anxiety, and reported that a significant decrease in postoperative anxiety. Although there was a difference between the 2 groups in the present study, 60% of the patients responded that listening to the surgeon’s postoperative explanations helped reduce their anxiety. This study, going beyond from the results of previous studies, demonstrated that postoperative explanations, such as postoperative pain or rehabilitation, are important for patients with anxiety regarding their postoperative course.

### Limitations

The present study had some limitations, the first of which was its single-institution design; thus, the results should be generalized with caution. Second, although all patients underwent arthroscopic meniscectomy, the location and chronicity of meniscal tears varied. Third, all patients included in this study underwent surgery under general anesthesia without the opportunity to choose their preferred method of anesthesia. Further studies are needed to compare patient anxiety levels when local anesthesia, which is known to have a shorter recovery time, less postoperative pain, and similar patient satisfaction as general anesthesia [24], is used as the anesthesia method. Fourth, there was a significant difference between the 2 groups with regard to sex, a limitation that can lead to statistical bias in the analysis of variation between the 2 groups. To confirm that statistical bias that may occur due to the high proportion of females in group I was not significant, we further analyzed the mean age between the sexes. Based on these results, we conclude that the 2 factors (female sex and age) were not confounding one another but were relatively independent risk factors. Finally, there was a wide range of time periods from the end of each surgery to the surgeon’s explanation. da Assunção et al. [25] reported a correlation between the interval between surgery and information delivery and patient recall of information regarding surgery. At our institution, postoperative ward rounds are initiated after all surgeries are completed on the same day. Depending on the order of surgeries, some patients could have heard the surgeon’s explanation within 1 h after surgery, whereas others could only meet with the surgeon several hours after the surgery was completed.

## Conclusions

Surgeons should be aware that anxiety related to arthroscopic partial meniscectomy differs according to patient characteristics, and a preoperative explanation of the postoperative process with the surgeon is important for patients who experience preoperative anxiety regarding anesthesia or the surgery itself.

## Data Availability

The datasets used and/or analyzed during the current study are available from the corresponding author on reasonable request.

## List of abbreviations

STAI: State-Trait Anxiety Inventory

## References

[1] Brattwall M, Jacobson E, Forssblad M, Jakobsson J (2010). Knee arthroscopy routines and practice. Knee Surg Sports Traumatol Arthrosc.

[2] Park SH, Jung KH, Chang SW, Jang SM, Park KB (2020). Trends in knee Surgery research in the official journal of the Korean Knee Society during the period 1999–2018: a bibliometric review. Knee Surg Relat Res.

[3] Forssblad M, Jacobson E, Weidenhielm L (2004). Knee arthroscopy with different anesthesia methods: a comparison of efficacy and cost. Knee Surg Sports Traumatol Arthrosc.

[4] Friberger Pajalic K, Turkiewicz A, Englund M (2018). Update on the risks of Complications after knee arthroscopy. BMC Musculoskelet Disord.

[5] Sertcakacilar G, Yildiz GO, Bayram B, Pektas Y, Cukurova Z, Hergunsel GO (2022). Comparing preoperative anxiety effects of Brachial Plexus Block and General Anesthesia for Orthopedic Upper-Extremity Surgery: a Randomized, Controlled Trial. Med (Kaunas).

[6] Baron JE, Khazi ZM, Duchman KR, Wolf BR, Westermann RW (2021). Increased prevalence and Associated costs of Psychiatric comorbidities in patients undergoing sports Medicine Operative procedures. Arthroscopy.

[7] Hines AC, Pill SG, Boes N, Reuschel B, Lutz A, Thigpen CA (2022). Mental health status, not resilience, influences functional recovery after arthroscopic rotator cuff repairs. J Shoulder Elbow Surg.

[8] Kaveeshwar S, Rocca MP, Oster BA (2022). Depression and anxiety are associated with worse baseline function in hip arthroscopy patients. Knee Surg Sports Traumatol Arthrosc.

[9] Park JH, Rhee SM, Kim HS, Oh JH (2021). Effects of anxiety and depression measured via the hospital anxiety and Depression Scale on Early Pain and Range of Motion after Rotator Cuff Repair. Am J Sports Med.

[10] Strube P, Schöpe T, Hölzl A, Brodt S, Matziolis G, Zippelius TK (2019). Influence of anxiety and Depression, Self-rated return-to-work problems, and unemployment on the outcome of Outpatient Rehabilitation after Shoulder Arthroscopy. Am J Phys Med Rehabil.

[11] Bayar A, Tuncay I, Atasoy N, Ayoğlu H, Keser S, Ege A (2008). The effect of watching live arthroscopic views on postoperative anxiety of patients. Knee Surg Sports Traumatol Arthrosc.

[12] Langford JL, Webster KE, Feller JA (2009). A prospective longitudinal study to assess psychological changes following anterior cruciate ligament reconstruction Surgery. Br J Sports Med.

[13] Yang JH, Ryu JJ, Nam E, Lee HS, Lee JK (2019). Effects of Preoperative Virtual Reality Magnetic Resonance Imaging on preoperative anxiety in patients undergoing arthroscopic knee Surgery: a randomized controlled study. Arthroscopy.

[14] Collimore KC, McCabe RE, Carleton RN, Asmundson GJ (2008). Media exposure and dimensions of anxiety sensitivity: differential associations with PTSD symptom clusters. J Anxiety Disord.

[15] Jung KH, Park JH, Song JY, Han JW, Park KB (2021). State-anxiety in geriatric patients undergoing Surgical Treatment for femoral Neck or Intertrochanteric fractures. Geriatr Orthop Surg Rehabil.

[16] Lee JS, Park YM, Ha KY, Cho SW, Bak GH, Kim KW (2016). Preoperative anxiety about spinal Surgery under general anesthesia. Eur Spine J.

[17] Cornoiu A, Beischer AD, Donnan L, Graves S, de Steiger R (2011). Multimedia patient education to assist the informed consent process for knee arthroscopy. ANZ J Surg.

[18] Tharion JG, Kale S (2021). Patient satisfaction through an immersive experience using a Mobile phone-based head-mounted Display during arthroscopic knee Surgery under spinal anesthesia: a Randomized Clinical Trial. Anesth Analg.

[19] Caumo W, Schmidt AP, Schneider CN, Bergmann J, Iwamoto CW, Banderira D (2001). Risk factors for preoperative anxiety in adults. Acta Anaesthesiol Scand.

[20] Jarmoszewicz K, Nowicka-Sauer K, Zemła A, Beta S (2020). Factors Associated with high preoperative anxiety: results from Cluster Analysis. World J Surg.

[21] Jlala HA, French JL, Foxall GL, Hardman JG, Bedforth NM (2010). Effect of preoperative multimedia information on perioperative anxiety in patients undergoing procedures under regional anaesthesia. Br J Anaesth.

[22] Soltner C, Giquello JA, Monrigal-Martin C, Beydon L (2011). Continuous care and empathic anaesthesiologist attitude in the preoperative period: impact on patient anxiety and satisfaction. Br J Anaesth.

[23] Sahin G, Basak T (2020). The effects of Intraoperative Progressive Muscle Relaxation and virtual reality application on anxiety, vital signs, and satisfaction: a Randomized Controlled Trial. J Perianesth Nurs.

[24] Barroso Rosa S, James D, Matthews BD (2016). Is knee arthroscopy under local anaesthetic a patient-friendly technique? A prospective controlled trial. Eur J Orthop Surg Traumatol.

[25] da Assunção RE, Neely J, Lochab J, Mizumi-Richards N, Barnett A, Pandit H (2013). Patient recall of surgical information after day case knee arthroscopy. Knee Surg Sports Traumatol Arthrosc.

